# Correction: The relationship between form and function throughout the history of excitation–contraction coupling

**DOI:** 10.1085/jgp.20171188901162018c

**Published:** 2018-02-05

**Authors:** Clara Franzini-Armstrong

Volume 150, No. 2, February 2018. https://doi.org/10.1085/jgp.201711889

The author regrets that in the original version of this article, Dr. Stephen Baylor’s first name was misspelled. Additionally, for Fig. 9 B, the hours postfertilization given in the legend was incorrect. The figure and its corrected legend appear below.

**Figure 9.**
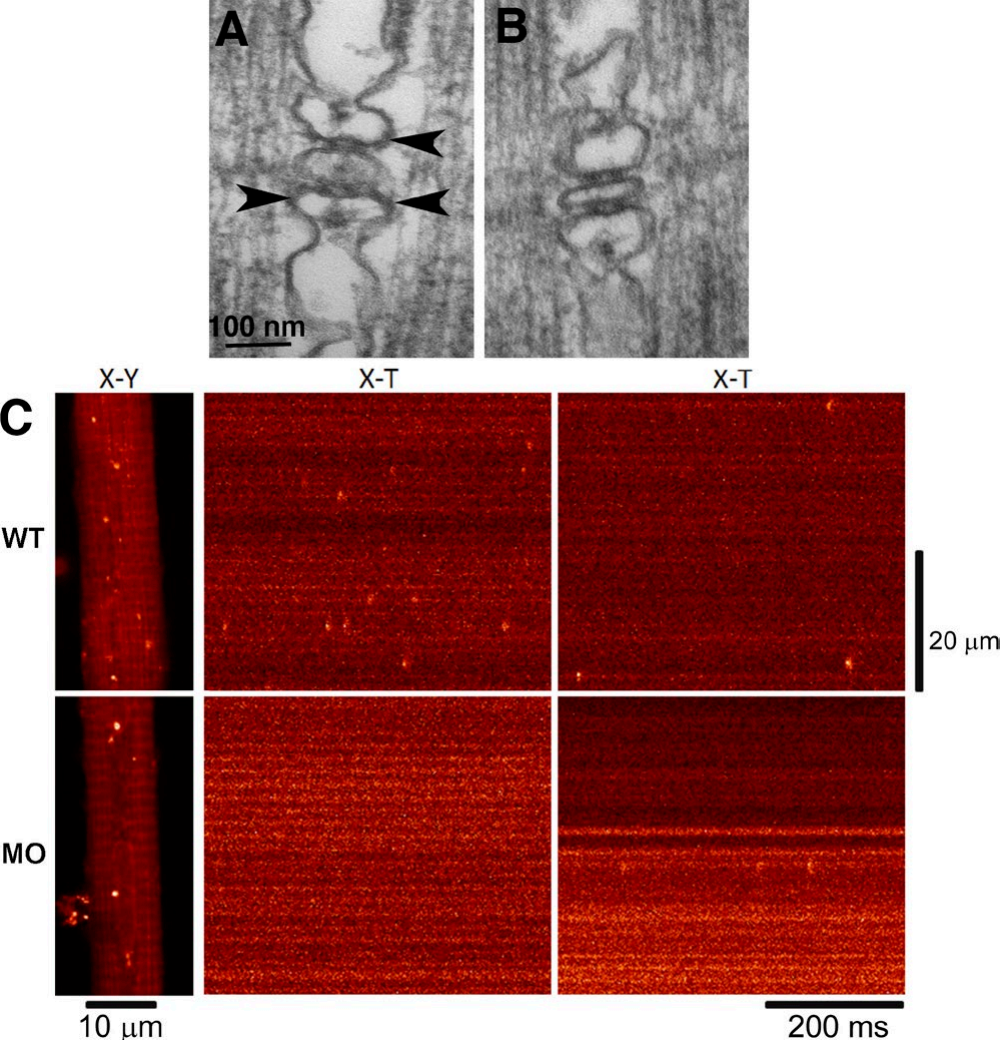
**RYR3 in a parajunctional position are necessary for the production of sparks in zebrafish muscle. (A)** Triad in a 72 h postfertilization larva. Two sets of feet (RYR1) connect SR to the central T tubule profile. Additional feet profiles in a parajunctional position (arrowheads) have been proposed to be RYR3 (Felder and Franzini-Armstrong, 2002). **(B)** One-cell-stage embryos were injected with a morpholino designed to specifically silence RYR3 expression. In triads of larvae at 72 h postfertilization, RYR1 position was normal, but parajunctional feet were essentially missing. **(C)** The Ca^2+^ sparks frequency in WT and morpholino-injected (MO) embryos dropped in correspondence to the absence of parajunctional feet. Reprinted from Perni et al. (2015).

All versions of this article have been corrected.

